# Diffusion Tensor Magnetic Resonance Imaging of Trigeminal Nerves in Relapsing Herpetic Keratouveitis

**DOI:** 10.1371/journal.pone.0122186

**Published:** 2015-04-01

**Authors:** Antoine Rousseau, Ghaïdaa Nasser, Christophe Chiquet, Emmanuel Barreau, Gael Gendron, Godefroy Kaswin, Mohamed M’Garrech, Farida Benoudiba, Denis Ducreux, Marc Labetoulle

**Affiliations:** 1 Department of Ophthalmology, Bicêtre Hospital, Assistance Publique—Hôpitaux de Paris, Paris-Sud University, Le Kremlin-Bicêtre, France; 2 Department of Neuroradiology, Bicêtre Hospital, Assistance Publique—Hôpitaux de Paris, Paris-Sud University, Le Kremlin-Bicêtre, France; 3 Department of Ophthalmology, Grenoble University Hospital, Grenoble, France; Brighton and Sussex Medical School, UNITED KINGDOM

## Abstract

**Background:**

Corneal hypoesthesia is the landmark of HSV and VZV keratitis and can lead to neurotrophic keratitis. Diffusion tensor imaging (DTI) is a new magnetic resonance imaging (MRI) derived technique, which offers possibilities to study axonal architecture. We aimed at assessing the potential impact of recurrent HSV or VZV-related keratitis on the axonal architecture of trigeminal nerves using DTI.

**Design:**

Prospective non-interventional study.

**Participants:**

Twelve patients and 24 controls.

**Methods:**

DTI using MRI of the trigeminal fibers and corneal esthesiometry using the Cochet-Bonnet esthesiometer were acquired for patients affected by unilateral and recurrent HSV or VZV-related keratitis (3 months after the last corneal inflammatory event), and control subjects with no history of ocular or neuronal disease affecting the trigeminal pathways.

**Main Outcome Measures:**

Fractional anisotropy (FA) and apparent diffusion coefficient (ADC) were compared between the 2 eyes of both patients and controls, and correlated with corneal esthesiometry.

**Results:**

FA was lower in the trigeminal fibers ipsilateral to the affected eye compared to the non-affected side (0.39±0.02 versus 0.46±0.04, P=0.03). This difference was more important than the intra-individual variability observed in controls. Concomitantly, the asymmetry in ADC results was significantly correlated with the loss of corneal sensitivity in the affected eye.

**Conclusions:**

Corneal hypoesthesia related to HSV and VZV keratitis is associated with persistent modifications in the architecture and functionality of the trigeminal fibers. These results add further explanation to the pathogenesis of HSV and VZV-induced neurotrophic keratitis, which may occur despite an apparent quiescence of the disease.

## Introduction

Herpes simplex virus (HSV) and varicella-zoster virus (VZV) share multiple pathogenic and clinical characteristics, such as the ability to become latent and to reactivate in trigeminal ganglion (TG) neurons, then to propagate through trigeminal sensory pathways before replicating in the eye, mostly in the cornea.

Both HSV and VZV infections are widely distributed in the general population: in middle-aged people, the seroprevalence is approximately 60% for HSV-1, and more than 95% for VZV in non-vaccinated subjects [1, 2]. The frequency of latent infection in the TG may be even higher, since modern biological methods have shown that 90 to 100% of people over 60 years of age have HSV1 and VZV DNA in their TG [3–6].

For HSV-seropositive patients, the lifetime risk of developing herpetic keratitis is 1%, with a relapse rate of 40% within 5 years after the first episode. This leads to a final incidence of herpetic keratitis comprised between 20 and 30 new events per 100,000 inhabitants annually [1, 7, 8]. Consequences of recurrent herpetic ocular disease are potentially sight-threatening, with up to 60% of deep corneal infections (stromal keratitis) inducing a visual acuity below 20/40 at 5 years of follow-up [9]. Concerning VZV seropositive patients, the lifetime risk of herpes zoster ophthalmicus (HZO) is about 1 to 2%, with corneal involvement in 65% of cases [10].

One of the most severe complications of HSV and VZV keratitis is the loss of corneal sensitivity. Corneal hypoesthesia leads to ocular dryness, epithelial defects [11, 12] and ultimately to neurotrophic keratitis, which occurs in up to 25% of HZO with corneal involvement [10, 13, 14].

While impairment of the corneal sensitivity is a well-known clinical fact, its underlying mechanisms are still questioned. It is known that viral latency occurs in the soma of the in trigeminal neurons (i.e. in the TG). When viral reactivation occurs, new particles of infectious HSV and VZV use anterograde transport from the TG to the cornea, along the trigeminal fibers (ophthalmic division). The resulting inflammation may explain the functional impairment of trigeminal termination in the cornea, but there is, to the best of our knowledge, no *in vivo* clear data showing that trigeminal fibers could be modified in their structure following recurrent HSV or VZV keratitis. *In vivo* confocal microscopy (IVCM) has shown significant alterations of the corneal sub-basal nerve plexus [15–17], however it does not exclude a functional and/or structural injury of the neuronal fibers within the trigeminal nerves. Corneal hypoesthesia may thus be secondary to either modifications of corneal receptors and/or neuronal fibers mediating the signal to the TG. Conventional computerized tomography (CT scan) and usual magnetic resonance imaging (MRI) are not precise enough to address this question since they underestimate nerve injuries [18]. Diffusion tensor imaging (DTI) is a non-invasive and non-ionizing functional imaging method based on a special MRI data processing. This technique allows an analysis of neuronal architecture through a complete description of water diffusion. In recent years, it has become a valuable technique to study traumatic axonal injury [18], and cranial nerve pathways anatomy and diseases, especially in the field of trigeminal nerve lesions [19–31].

Two main parameters can be studied with DTI: the fractional anisotropy (FA), which reflects the water diffusivity along the axonal axis and thus might assess the integrity of the tissues, and the apparent diffusion coefficient (ADC), which is related to the water exchange between the extracellular and intracellular compartments. Moreover, several studies in other fields of interest have shown that the level of axonal injury is associated with FA reduction and changes of the ADC. Indeed, decreased ADC values reflect intra-cellular edema (cytotoxic edema) and increased ADC values correspond to an increase in the extra-cellular space (vasogenic edema) [18, 32–34]. To accurately assess the potential impact of trigeminal fibers damages in the loss of corneal sensation secondary to severe viral corneal disease, we used DTI in patients with unilateral keratitis or keratouveits (KU) related to HSV or VZV, either biologically proved or strongly suspected on the basis of clinical history. To the best of our knowledge, this is the first study of a structural analysis of the trigeminal pathways in HSV and VZV induced corneal hypoesthesia.

## Methods

This study was approved by the national institutional review board (Ethics Committee of the French Society of Ophthalmology) and was conducted in accordance with the tenets of the Declaration of Helsinki. Informed consent was obtained orally for all the subjects enrolled in the study, and recorded in medical files by the physician in charge of the patient. As MRI is a safe and non invasive technology to assess trigeminal dysfunction [35], written research consent was not considered as mandatory. However, all participants signed an informed consent form explaining the technical issues of MRI prior to the examination.

### Patients

All the patients referred to our department for a proven or strongly presumed VZV/HSV-related keratitis or KU between September 2008 and September 2009, were prospectively analyzed for inclusion in this study. The clinical diagnosis of keratitis or KU was made upon slit lamp findings (corneal swelling, keratic precipitates, and flare in the anterior chamber). The VZV etiology was suspected on a recent history of HZO with ocular involvement, either during the acute phase of the vesicular eruption or delayed. The HSV-related ocular disease was suspected on: i) a previous history of multiple relapses of keratitis or KU, ii) the efficacy of anti-herpetic drugs (oral valaciclovir, oral or topical acyclovir, topical ganciclovir, trifluridine) as curative or preventive treatment for previous episodes and iii) no history of herpes zoster (whatever the location). In cases of KU, anterior chamber taps were performed. The presence of HSV and VZV in aqueous humor (AH) was evaluated by polymerase chain reaction (PCR) or by the Witmer-Goldmann coefficient in paired AH and serum samples (considered positive if greater than 2). All the patients were examined and diagnosed by the same ophthalmologist (ML). Clinical and biological data were anonymously recorded from clinical files. The corneal sensitivity was tested in central cornea, with a Cochet-Bonnet contact esthesiometer (CBE) (Luneau, France), as previously described [36]. This choice was supported by the fact that more than 60% of HSV-induced hypoesthesia are detectable in the central cornea [37].

Patients finally selected for the study were those with clinical signs of keratitis or KU, a typical past history of VZV or HSV keratitis or KU, and/or a positive test in AH. The DTI was planned at least 3 months after the last examination showing a resolution of acute keratitis or KU.

### Control subjects

As older subjects have axonal loss because of the physiological white matter atrophy process [38], age-matched control subjects were included for reference values, in a 2:1 ratio with keratitis/KU patients. They were either volunteers (staff of the neuroradiology department) or patients who underwent imaging for a disease with no presumed damage of the trigeminal system. None of these control subjects had a history of herpes recurrences or HZO, and MRI was used for DTI processing if no evidence of central nervous system disease was observed.

### Diffusion tensor imaging

Investigations were performed on a 1.5T system (Sonata scanner, Siemens, Erlangen, Germany). The MRI protocol consisted of an axial 3D T1-weighted scan (TR/TE, 11/4 ms), a FLAIR scan (TR/TE/TI, 9480/112/2390 ms), an axial T*-weighted GE scan (TR/TE, 1330/33 ms), and an axial echo-planar imaging (EPI) DTI scan (TR/TE, 5700/110 ms; FOV, 24x24 cm; image matrix, 128x128; number of sections, 30; section thickness, 2 mm; nominal voxel size, 1.875x1.875x2 mm; number of signal intensity averages, 3) with diffusion gradients set in 25 non-collinear directions by using 2 b-values (b = 0 and 500 s/mm2) to minimize the signal loss due to the strong diffusion gradients. The DTI scanning took approximately 7 minutes 30 seconds. The DTI data were processed on a voxel-by voxel basis with dedicated software (DPTools [http://www.fmritools.org]).

A correction algorithm was applied to the DTI dataset to account for distortions that were related to eddy currents induced by the large diffusion-sensitizing gradients. It relied on a 3-parameter distortion model including scale, shear, and linear translation in the phase-encoding direction, as previously described [18]. A single physician (FB) carried out the MRI acquisition. Image analysis was performed by another physician (GN) who was masked for the group (patient or control) and for the side affected by the herpetic ocular disease (if applicable).

**Fig 1:**
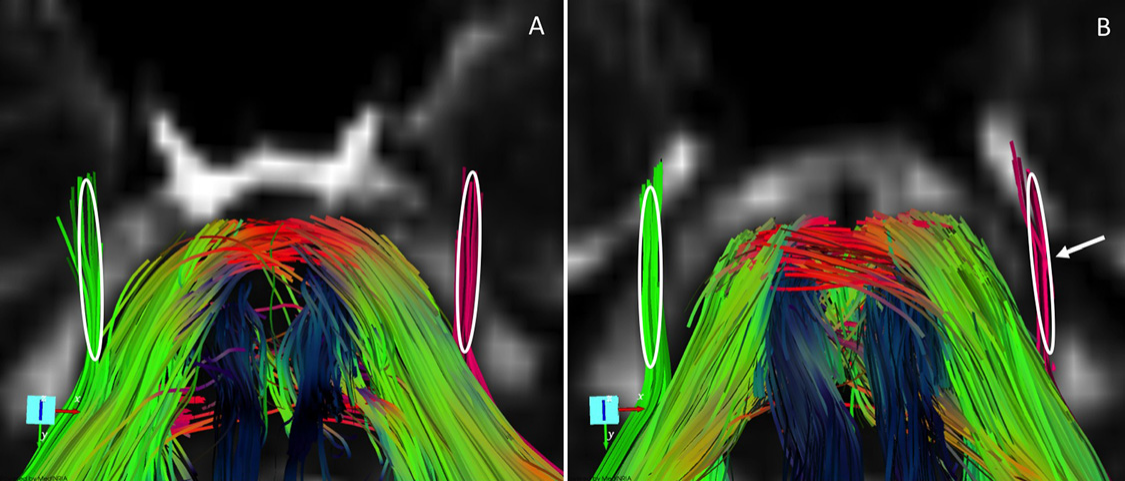
Tractography and delineation of regions of interest (white circles) of trigeminal nerves in a control (control n° 1, image A) and a patient with a left herpetic stromal keratitis (patient n°2, image B). These images illustrate the cisternal portion of the trigeminal nerves. The fibers are colour-coded for side: red fibers are on the left, green fibers are on the right). In the control, trigeminal nerves are symmetric. Note the reduction of the number of fibers in affected side (left) of the patient (arrow).

Fiber tracking was performed with dedicated software (MedINRIA[http://www-sop.inria.fr/asclepios/software/MedINRIA]). White matter fiber tracts were created in 3D on the basis of similarities between neighboring voxels in shape (quantitative diffusion anisotropy measures) and orientation (principal eigenvector map) of the diffusion ellipsoid and co-registered on the FA map by using a special algorithm previously described [39, 40]. The principal diffusion direction method [40–42] was used, in which the eigenvector corresponding to the largest eigenvalue is extracted from the diffusion tensor field generated from the DTI datasets in the region where the diffusion is linear. The FA threshold value was 0.20; and the angulation threshold, 45° to prevent fibers from sudden transition and to keep tracking based on the connectivity of the neighborhood, as described elsewhere [40, 42]. The 3D fiber reconstructions were color-coded so that blue represented the superior-inferior; green, the anteroposterior; and red, the left-right direction. Tractography was used to locate the trigeminal pathways. Regions of interest of 1.875×1.875×2 (x×y×z) voxels (total = 12 voxels = 84mm^3^) were manually drawn on the reconstructed fibers, in order to include all the visible part of the extra-axial emerging trigeminal fibers (fig. 1). As there are many crossing fibers in the intra-axial trigeminal nerve and near the root entry transitional zone, measurements in these regions may have lead to important partial volume effect.

Data were anonymously recorded before processing to retrospective statistical analysis using STATA software (Statacorp LP, USA). Categorical variables were analyzed using the χ^2^ test and continuous variables were analyzed using the non-parametric Kruskall-Wallis test for mean comparison and non-parametric Spearman test for correlation. Statistical significance was defined as *P* < 0.05 (2-tailed).

## Results

Twelve consecutive patients (5 males and 7 females) with a proven or strongly presumed VZV/HSV-related keratitis or KU, according to the inclusion criteria, were selected. Their mean age at time of DTI was 56.4±9.1 years, and the mean age at the first episode of viral keratitis was 43.5±10.6 years (Table 1). The ocular disease was unilateral in 100% of cases (affected side: 10 left, 2 right). At the time of DTI procedure, the mean visual acuity of affected eyes was 0.84±0.29 versus 0.94±0.10 in the non-affected eye. Eleven patients (92%) were treated by a long-term preventive regimen of either valaciclovir (9 patients) or acyclovir (2 patients). Anterior chamber taps were performed at the time of ongoing intraocular inflammation in 8 patients (67%), PCR or Witmer-Goldman coefficient was positive for either HSV1 or VZV in 5 patients (Table 1). For the 4 remaining patients, no aqueous tap had been performed, either because they presented with a pure stromal keratitis (i.e. with no sign of anterior chamber inflammation) and a previous history of similar events resolving with antiviral medications, or because keratitis developed in the weeks following a HZO.

**Table 1:** Summary of the clinical data of the patients with a proven or strongly presumed keratitis or keratouveitis related to either HSV or VZV.

Patient n°	Sex	Age at onset (yrs)	Age at DTI (yrs)	Delay crisis/DTI (mths)	Affected Eye	Clinical Aspect	Virus	Type of evidence	Affected eye esthesiometry (mm)	Non-affected eye esthesiometry (mm)
1	M	60	71	5	L	KU	HSV	Clinical	25	50
2	F	23	43	3	L	K	HSV	Clinical	15	40
3	M	67	69	3	L	KU	HSV	Clinical	35	40
4	M	53	61	99	L	KU	VZV	PCR in AH	50	55
5	M	41	44	30	L	K	HSV	Clinical	20	40
6	F	17	54	16	L	K	HSV	WG in AH	20	40
7	M	60	69	65	L	KU	HSV	PCR in AH	30	45
8	F	32	71	60	L	K	HSV	Clinical	25	55
9	F	74	78	12	R	KU	VZV	PCR in AH	35	55
10	F	24	26	5	L	K	HSV	Clinical	20	55
11	F	36	53	3	R	KU	HSV	Clinical	45	55
12	F	35	38	18	L	KU	HSV	PCR in AH	35	55
Mean		43.5	56.4						29.6	48.7
SE		10.6	9.1	17.8					6.1	4.0

Abbreviations: AH: aqueous humor, DTI = diffusion tensor imaging, K = keratitis KU = keratouveitis, mths = months, ND: not done, PCR = polymerase chain reaction, SE = standard error, WG = Witmer-Goldman coefficient, yrs = years.

Twenty-four control subjects were included. The mean age was 58.1±6.1 years and 11 were males. Patients and control subjects were comparable in terms of age and sex-distribution (*P* = 0.80 and *P* = 0.81, respectively).

**Fig 2:**
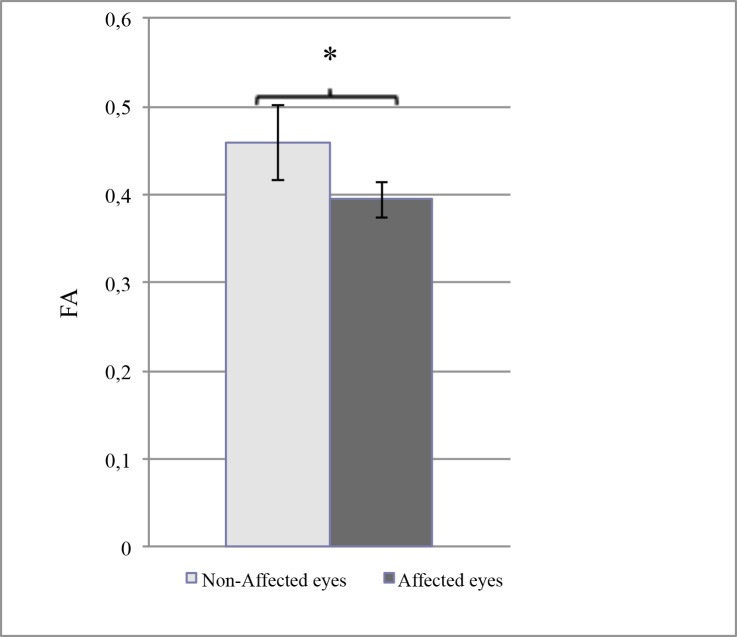
Comparison between the fractional anisotropy (FA) results in the affected eyes and the in non-affected eyes of the patients with a proven or strongly presumed keratitis or keratouveitis related to either HSV or VZV. The bars indicate the standard error of the mean. Asterisk shows that the difference between values was significant (Kruskal-Wallis test, *P* = 0.03).

**Fig 3:**
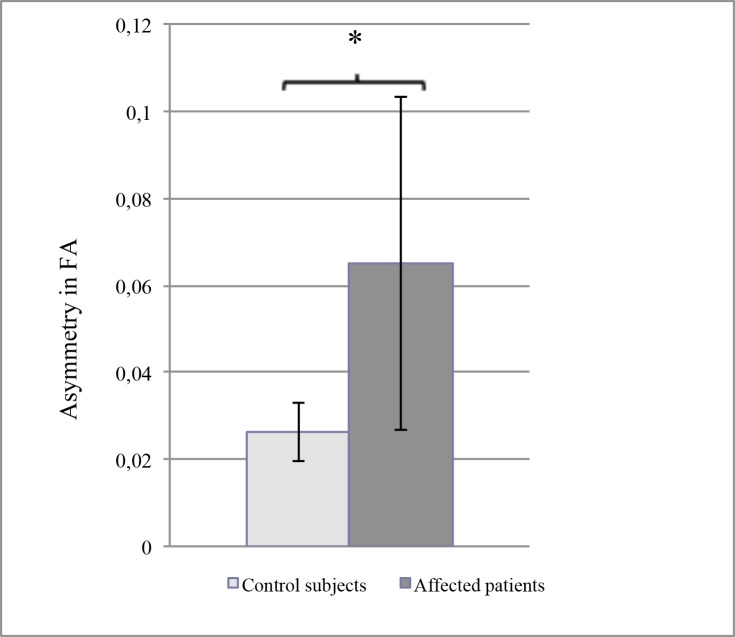
Comparison between the asymmetry of fractional anisotropy (FA) results in patients with a proven or strongly presumed keratitis or keratouveitis related to either HSV or VZV (non-affected eye minus affected eye) and in the control group (higher value minus lower value). The bars indicate the standard error of the mean. Asterisk shows that the difference between values was significant (Kruskal-Wallis test, *P* = 0.02).

**Fig 4:**
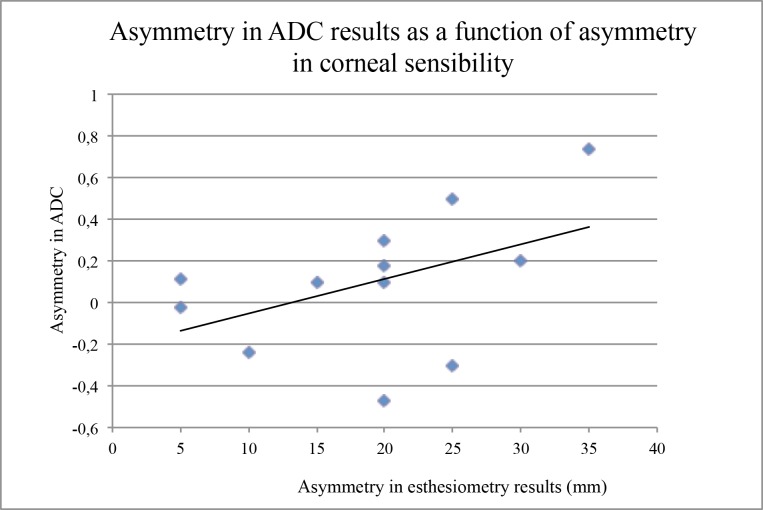
Asymmetry in the corneal sensitivity (affected eye minus non-affected eye) as a function of the asymmetry in apparent diffusion coefficient (ADC) results (higher value minus lower value) in patients with a proven or strongly presumed keratitis or keratouveitis related to either HSV or VZV. The solid line indicates the significant correlation between results (Spearman test, *P* = 0.05).

The results of DTI, performed with a mean delay of 26.6±17.7 months after the last acute ocular episode in the keratitis group, are summarized in Table 2 (details in Tables 3 and 4). In the control group, there was no significant difference between the right and the left trigeminal pathways concerning the FA values (0.40±0.03 versus 0.39±0.02, *P* = 0.57) and the ADC results (1.69±0.10 vs 1.66±0.09, *P* = 0.68). In contrast, in the keratitis group, the FA results were significantly lower in the trigeminal fibers ipsilateral to the affected eye than in those of the non-affected side (0.39±0.02 versus 0.46±0.04, *P* = 0.03) (Fig. 2). Moreover, the asymmetry between the affected and non-affected side (mean difference: 0.06±0.03) was significantly more important than the mean difference between all higher and all lower values in control subjects (0.02±0.01, *P* = 0.02) (Fig. 3). However, such a significant difference between affected and non-affected side was not observed for the ADC values (1.81±0.14 vs 1.72±0.2, *P* = 0.32), and the mean asymmetry in these patients was comparable to the maximal asymmetry assessed in control subjects (0.1±0.19 vs 0.11±0.03, *P* = 0.8). Since ADC is known as a marker of the quality of water exchange in the nerve fibers, and thus of their normal physiology, we compared these results with those of the corneal sensitivity, using the CBE. As expected, there was a significant asymmetry between affected versus non-affected eyes (29.6±6.0 mm versus 48.7±4.0 mm, *P* = 0.0003) in patients presenting a recurrent or severe corneal disease due to HSV or VZV. When the difference in corneal sensitivity was compared to the difference (asymmetry) in FA results, no relationship was observed among the patients, but a significant correlation (*P* = 0.05, Spearman test) was observed between the loss of corneal sensitivity and the asymmetry in ADC results (Fig. 4).

**Table 2:** Summary of the trigeminal fibers tracking using Diffusion Tensor Imaging in patients and control subjects.

	Keratitis group Mean values (±SE)	Control group Mean values (±SE)	P value
**Age**	56.4 (±9.1)	58.2 (±6.2)	
**Sex-ratio (M/F)**	5/7	11/13	
**FA in affected eyes**	0.39 (±0.02)		
**FA in non-affected eyes**	0.46 (±0.04)		***P = 0*.*03***
**FA asymmetry in patients (Affected versus non-affected eyes)**	0.06 (±0.03)		
**ADC in affected eyes**	1.81 (±0.14)		
**ADC in non-affected eyes**	1.72 (±0.20)		*P = 0*.*32*
**ADC asymmetry in patients (Affected versus non-affected eyes)**	0.10 (±0.19)		
**FA in right eye**		0.40 (±0.03)	
**FA in left eye**		0.39 (±0.02)	*P = 0*.*57*
**Difference in FA in left versus right eyes**		0.01 (±0.01)	*P = 0*.*17*
**Lower FA**		0.39 (±0.02)	
**Higher FA**		0.41 (±0.02)	*P = 0*.*10*
**Difference in FA between higher and lower results**		0.02 (±0.01)	
**ADC in right eye**		1.69 (±0.10)	
**ADC in left eye**		1.66 (±0.09)	*P = 0*.*68*
**Difference in ADC in left versus right eyes**		-0.02 (±0.06)	*P = 0*.*17*
**Lower FA**		0.39 (±0.02)	
**Higher FA**		0.41 (±0.02)	*P = 0*.*10*
**Difference in FA between higher and lower results**		0.02 (±0.01)	
**ADC in right eye**		1.69 (±0.10)	
**ADC in left eye**		1.66 (±0.09)	*P = 0*.*68*
**Difference in ADC in left versus right eyes**		-0.02 (±0.06)	*P = 0*.*60*
**Lower ADC**		1.62 (±0.08)	
**Higher ADC**		1.73 (±0.10)	*P = 0*.*12*
**Difference in ADC between higher and lower results**		0.10 (±0.09)	
**FA in affected eyes**	0.39 (±0.02)		
**Lower FA in control eyes**		0.39 (±0.02)	*P = 0*.*46*
**ADC in affected eyes**	1.81 (±0.14)		
**Higher ADC in control eyes**		1.73 (±0.10)	*P = 0*.*42*
**FA asymmetry in patients**	0.06 (±0.03)		
**Maximal FA asymmetry in controls**		0.02 (±0.01)	***P = 0*.*02***
**ADC asymmetry in patients**	0.10 (±0.19)		
**Maximal ADC asymmetry in controls**		0.10 (±0.03)	*P = 0*.*80*

Abbreviations: ADC = apparent diffusion coefficient, FA = fractional anisotropy, SE = standard error.

**Table 3:** Details of the diffusion tensor imaging results of the 12 VZV or HSV-related keratitis or keratouveitis patients.

			Right side		Left Side	
Patient n°	Sex	Age (yrs)	FA mean	ADC mean	FA mean	ADC mean
1	M	71	0.33	2.40	0.38	2.10
2	F	43	0.49	1.40	0.43	1.90
3	M	69	0.5	1.90	0.42	1.88
4	M	61	0.5	1.49	0.40	1.60
5	M	44	0.48	1.60	0.41	1.78
6	F	54	0.54	2.20	0.36	2.30
7	M	69	0.49	1.40	0.45	1.50
8	F	71	0.57	1.30	0.38	1.50
9	F	78	0.37	2.00	0.41	1.70
10	F	26	0.48	1.30	0.44	2.04
11	F	53	0.35	1.60	0.37	1.84
12	F	38	0.35	2.07	0.34	1.60
Mean		56.42	0.45	1.72	0.40	1.81
SE		9.10	0.05	0.21	0.02	0.14

Note: ADC indicates apparent diffusion coefficient; FA, fractional anisotropy; SE, standard error; yrs, years.

**Table 4:** Details of the diffusion tensor imaging results of the 24 control patients (sex- and age-marched volunteers or patients with normal MRI, no previous history of herpetic or zoster disease).

			Right side		Left Side	
Control n°	Gender	Age (yrs)	FA mean	ADC mean	FA mean	ADC mean
1	F	38	0.4	1.40	0.44	1.50
2	F	24	0.49	1.58	0.52	1.56
3	M	59	0.48	1.50	0.45	1.60
4	F	79	0.37	1.70	0.37	1.68
5	M	71	0.49	1.56	0.45	1.60
6	M	37	0.5	1.69	0.45	1.80
7	F	70	0.4	1.50	0.39	1.49
8	F	43	0.34	2.20	0.32	1.80
9	F	69	0.49	1.20	0.45	1.30
10	F	81	0.54	1.60	0.48	1.40
11	F	60	0.33	2.00	0.33	2.20
12	F	54	0.33	1.70	0.37	1.80
13	M	36	0.48	1.30	0.45	1.29
14	F	52	0.38	1.70	0.38	1.70
15	F	59	0.36	1.90	0.38	1.70
16	M	78	0.38	1.35	0.43	1.50
17	M	72	0.35	2.10	0.33	1.90
18	M	78	0.32	1.98	0.34	1.87
19	F	48	0.37	1.80	0.38	1.60
20	M	52	0.36	1.70	0.33	1.70
21	M	55	0.37	1.80	0.36	1.96
22	M	54	0.38	1.70	0.35	1.60
23	M	61	0.39	1.90	0.37	1.90
24	F	66	0.38	1.60	0.35	1.50
Mean		58.17	0.40	1.69	0.39	1.66
SE		6.13	0.03	0.10	0.02	0.09

Note: ADC indicates apparent diffusion coefficient; FA, fractional anisotropy; SE, standard error; yrs, years.

## Discussion

The number and the frequency of recurrent HSV keratitis are the main risk factors of persistent and deep loss in corneal sensitivity [37]. Thus, recurrent history of stromal inflammation due to HSV keratitis has been shown to be associated with neuropathic pain, discomfort, ocular dryness and corneal hypoesthesia [43]. Some stromal inflammation may also persist in 30% of patients affected by HZO [12], which could explain that a single episode of HZO can result in a significant diminution of corneal sensitivity with subsequent loss of corneal epithelial integrity and lacrimal dysfunction, leading in some patients to neurotrophic keratitis.

In patients with HSV or VZV keratitis, the reduction of corneal sensitivity is of variable magnitude, mainly affecting mechanical and heat sensation [44, 45]. Loss of corneal sensitivity has been correlated with sub-basal nerve plexus alterations (i.e. decrease in nerve density and nerve number), as shown by IVCM [15–17]. However, these lesions could reflect a more diffuse wallerian-type degeneration of the trigeminal axons. DTI provides an *in vivo* evaluation of trigeminal fibers that had not been previously conducted in herpes simplex and herpes zoster eye disease. Our results show that the recurrent/chronic corneal infection by HSV or VZV results in persistent modifications of the trigeminal fibers. A possible explanation could be an impaired neurotransmission by trigeminal neurons consecutively to the HSV latent infection. Indeed, experiments in mice showed altered neuronal expression of genes encoding potassium voltage-gated channels and a muscarinic acetylcholine receptor [46]. Moreover, *in vitro* HSV-infected sensory neurons showed modification of voltage channels [47].

It is not known whether the microstructural changes of the affected TG are homogeneous or not: there could be focal variations of FA and ADC values in the affected TG. However, this potential heterogeneity seems difficult to assess with DTI. Indeed, ROI were limited to the visible part of the extra-axial emerging trigeminal fibers because measurements in the intra-axial trigeminal nerve and near the root entry transitional zone may have lead to important partial volume effect. These ROI are very small and cannot be divided into sub-ROI that would have been required to demonstrate some heterogeneity.

Compared to the contralateral asymptomatic side, trigeminal fibers were altered on the affected side, as suggested by the significantly reduced FA. Indeed, FA may be a good marker of trigeminal nerve integrity, even if, to the best of our knowledge, there are no published data on histologic/DTI correlations in injured trigeminal nerves.

Interestingly, in an experimental study, DTI was applied to a murine model of Krabbe disease to explore relations between DTI and spinal cord histological changes. Hofling et al. found that FA was significantly decreased in the spinal cord where they histologically demonstrated axonal injury and demyelination. Unfortunately they did not study the histological changes in the trigeminal nerves where FA was also significantly decreased [30]. In the clinical setting, Hodaie et al. found that FA dropped significantly (47%) in the trigeminal nerves after radiosurgical treatment for trigeminal neuralgia. In the same study, recovery of FA in the treated portion of the trigeminal nerves correlated with pain recurrence [28]. In patients with trigeminal neuralgia, Liu et al. found that compared with the unaffected side and healthy controls, the affected trigeminal nerve showed significantly decreased FA and there was a significant correlation between the FA reduction and pain, assessed with a visual analogy scale [31].

Additionally, we observed that the level of asymmetry in ADC results between affected and non-affected side was correlated with the level in the loss of corneal sensitivity in the affected cornea. Altogether, our results suggest that HSV and VZV keratitis induce significant alterations of the trigeminal fibers that are still present several months after the last episode of ocular inflammation.

We also noticed that the values of CBE in the unaffected fellow eyes of patients (48.7mm±4) were lower than those previously described in normal eyes (59mm±0.2) [36]. This is in keeping with the results of many recent studies showing either a decrease in corneal sensitivity or ocular dryness in unaffected fellow eyes of patients with unilateral anterior herpetic eye disease [43, 45]. In a recent study, we also demonstrated that the decrease in corneal sensation and the impairment of lacrimal function of the fellow unaffected eye are correlated with the type/severity of the HSV or VZV keratitis [48]. In addition, using IVCM, Hamrah et al. showed that corneal nerves are damaged in the contralateral eye of both HSV and VZV keratitis [15, 16]. Interestingly, most of PCR results in human tissues found HSV or VZV genome in the both TG despite the unilateral nature of the clinical diseases [3–6]. Moreover, a unilateral inoculation of HSV leads to bilateral TG latent infection in mice [49]. However, using DTI, we were not able to demonstrate significant alterations of the trigeminal pathways of the unaffected eye. It is possible that a larger number of subjects enrolled and more sensitive imaging systems such as 3 Tesla MRI could provide additional results and conclusions.

Histopathologic studies of ganglia undergoing the reactivation process of VZV revealed inflammation, necrosis, and disruption of the morphology of neuronal and non-neuronal cells [50]. A similar inflammation with nerve damage, and possible demyelization, is believed to occur in the trigeminal system following the replication of HSV, as suggested by several experimental studies [51–53]. In human, recent studies have clearly showed that despite the absence of clinical signs of ongoing HSV infection into the ocular tissues, some low-grade viral replication may persist in herpetic patients, as shown by the high rate of HSV shedding in the tears of asymptomatic patients [54]. Similarly, periodic episodes of subclinical VZV reactivation are known to occur in the trigeminal ganglia of VZV infected patients throughout their lifetime, serving as immune boosters that increase the cell-mediated immune response to VZV [12]. Furthermore, some viral DNA can still be present on the ocular surface several weeks after the onset of acute HZO and after chickenpox [55–58]. Finally, post-mortem human studies showed a chronic inflammation in the sensory system of patients with a history of HSV1 and VZV infection [59, 60].

Taken together, the previously published data and our results may provide an explanation for the fact that some neurotrophic keratitis become clinically significant several months after the acute phase of the disease, arguing for a continuous process of nerve injury due to ongoing inflammation related to continuous viral triggering. This point should be clarified by a prospective follow-up of patients using esthesiometry and DTI imaging over time.

As a conclusion, DTI permitted us to show that HSV and VZV unilateral corneal diseases are associated with both a loss of corneal sensitivity and a modification in the architecture and functionality of the trigeminal fibers connected to the affected eye. This could either reflect a sequel from the acute phase of the disease or an ongoing process due to continuous low-grade viral replication in the trigeminal neurons. In both cases, these data emphasize the need for preventive treatments with the aim of reducing the nerve injury, and thus the rate of late-stage corneal complications.
